# The Rho GTPase signaling pathway modulates *Moraxella catarrhalis* invasion into human respiratory epithelial cells by regulating actin polymerization

**DOI:** 10.3389/fimmu.2026.1730864

**Published:** 2026-02-17

**Authors:** Ruirui Ma, Guixue Cheng, Yun Wu, Jiawei Chen, Rongqi Lu, Yali Liu

**Affiliations:** 1 Department of Clinical Laboratory, State Key Laboratory of Complex Severe and Rare Diseases, Chinese Academy of Medical Sciences and Peking Union Medical College Hospital, Beijing, China; 2 Graduate School, Peking Union Medical College, Chinese Academy of Medical Sciences, Beijing, China; 3 Department of Clinical Laboratory, Shengjing Hospital of China Medical University, Shenyang, China

**Keywords:** F-actin, G-actin, invasion, *Moraxella catarrhalis*, Rho GTPase

## Abstract

*Moraxella catarrhalis* invasion of host respiratory epithelial cells is a critical mechanism driving acute exacerbations of chronic obstructive pulmonary disease (AECOPD). Although previous studies have extensively demonstrated that dynamic changes in the actin cytoskeleton are central to the invasion of host cells by *Moraxella catarrhalis*, the detailed mechanisms underlying the specific upstream signaling pathways and key regulators driving this process remain incompletely understood. Our study identifies and validates the essential roles of key Rho GTPase regulators (CDC42, Rac1, ArpC2, ArpC4) in actin polymerization during *M. catarrhalis* infection, thereby elucidating a more comprehensive and specific molecular mechanism. Invasion assays and Transmission electron microscopy (TEM) showed that the Rho GTPase signaling pathway modulates *M. catarrhalis* bacterial load in A549 cells by regulating macropinosome volume. Further experiments used *M. catarrhalis* strains 73-OR and ATCC 25238 to invade wild-type A549 cells, CDC42^-/-^ A549 cells, Rac1^-/-^ A549 cells, ArpC2^-/-^ A549 cells and ArpC4^-/-^A549 cells respectively. Invasion assays and TEM were performed to quantify internalized bacteria, macropinosome volume changes, and bacterial distribution; Western blot analyses and cellular immunofluorescence were used to measure F-actin/G-actin ratios and microfilament fluorescence intensity. These results indicate that Rho GTPase signaling pathway modulates *M.catarrhalis* invasion by regulating actin polymerization dynamics. Specifically, CDC42 and Rac1 are essential for actin polymerization and bacterial internalization. ArpC4 contributes to actin remodeling without influencing invasion, while ArpC2 is uninvolved in both processes. These findings provide a theoretical basis for targeting innate immunity to prevent and treat *M. catarrhalis*-induced AECOPD.

## Introduction

*Moraxella catarrhalis* (*M.catarrhalis*) is a non-encapsulated, non-flagellated Gram-negative diplococcus. It is a common colonizer of the human upper respiratory tract, predominantly inhabiting the nasopharynx. Colonization rates reach 5% in healthy adults but can escalate to 72–100% in healthy children (1–5). Since 1972, extensive research has established *M. catarrhalis* as a significant pathogen in clinical infections, including otitis media, sinusitis, lower respiratory tract infections, infective endocarditis, and bacteremia (1, 6). In adults with chronic obstructive pulmonary disease (COPD), *M.catarrhalis* is the second most common pathogen (after *Haemophilus influenzae*) implicated in acute exacerbations of COPD (AECOPD) (7). Among AECOPD patients, *M. catarrhalis* detection rates range from 10% to 20%, which correlates with increased hospitalization frequency, antibiotic usage, and healthcare economic burdens (8, 9). Recurrent *M. catarrhalis* infections induce chronic inflammatory states that disrupt pulmonary microenvironments and microbiota, accelerating AECOPD progression—a critical risk factor for mortality in these patients (10).

The invasion of human respiratory epithelial cells by *M. catarrhalis* is a critical step in initiating lower respiratory tract infections in patients with AECOPD. During the pathogenesis, *M. catarrhalis* interacts with host respiratory epithelial cells and then activates the immune response through various extracellular and intracellular receptors. Studies have found that *M. catarrhalis* invades host respiratory epithelial cells via macropinocytosis, a pathogenic mechanism shared by intracellular and extracellular bacteria. During macropinocytosis, the actin cytoskeleton of host respiratory epithelial cells first polymerizes from actin monomers (also known as globular actin or G-actin) into actin polymers (also known as filamentous actin or F-actin), and then assembles to form microfilaments. Microfilament extension can then form ruffles. Previous studies have demonstrated that cytochalasin D, an actin polymerization inhibitor that severs microfilaments and blocks actin addition to filament ends, significantly reduces *M. catarrhalis* invasion in BEAS-2B human lung epithelial cells (11). Our research in murine COPD models and *in vitro* experiments has confirmed that *M. catarrhalis* internalization into human alveolar type II epithelial cells is actin-dependent, and inhibition of actin polymerization effectively blocks macropinocytosis-mediated bacterial uptake (12). Therefore, actin polymerization/dissociation is essential for initiating and regulating the macropinocytosis process.

The actin cytoskeleton is a highly dynamic structure regulated by numerous proteins and molecular interactions, among which Rho GTPases play a crucial role. Rho GTPases are small molecule proteins in eukaryotes that mediate critical cellular processes, with over 20 members and more than 70 downstream effector proteins (13, 14). Among these, Rac1 promotes lamellipodia and membrane ruffle formation, while CDC42 drives filopodia formation. During bacterial invasion, Rac1 and CDC42 indirectly regulate actin polymerization to induce pseudopod formation, thereby modulating epithelial cell endocytosis of pathogens such as *Pseudomonas aeruginosa*, *Yersinia* spp., and *Clostridioides difficile* (13, 15). This study will further investigate the mechanisms by which Rho GTPases regulate actin polymerization and *M. catarrhalis* invasion into human respiratory epithelial cells, based on existing evidence. By identifying key regulatory factors in this process, we intended to provide a foundation for developing novel therapeutic strategies to prevent or treat *M. catarrhalis*-induced acute exacerbations of chronic obstructive pulmonary disease (AECOPD).

## Experimental methods

### Bacterial strains and reagents

This study was conducted from May 2024 to January 2025 at Chinese Academy of Medical Sciences and Peking Union Medical College*. M. catarrhalis* strain 73-OR, derived from prior studies, was a macrolide-sensitive clone belonging to clonal complex CC446 (lacking the A2330T mutation), which exhibits robust invasive capacity in human alveolar type II epithelial cells (A549) (16). The wild-type (WT) strain *M. catarrhalis* ATCC 25238 was purchased from the American Type Culture Collection (ATCC).

Actin-related protein 2/3 complex (Arp2/3) inhibitor CK-636 (CK-0944636, Cat. No. IC3650, Beijing Solarbio Science &Technology Co., Ltd), CDC42 GTPase inhibitor ML 141 (ab145603, Cat. No. 10155-1-AP, Proteintech Group, Inc. Chicago, USA), actin polymerization inhibitor: Latrunculin A (Cat. No. HY-16929, MedChem Express LLC), Rho GTPase inhibitor: Simvastatin (Cat. No. IS0170, Beijing Solarbio Science &Technology Co., Ltd.), Rac1/CDC42 activator: CN02-B (Cat. No. CN02-B, Cytoskeleton, Inc.).

### Cell lines and culture conditions

**Figure 1:**
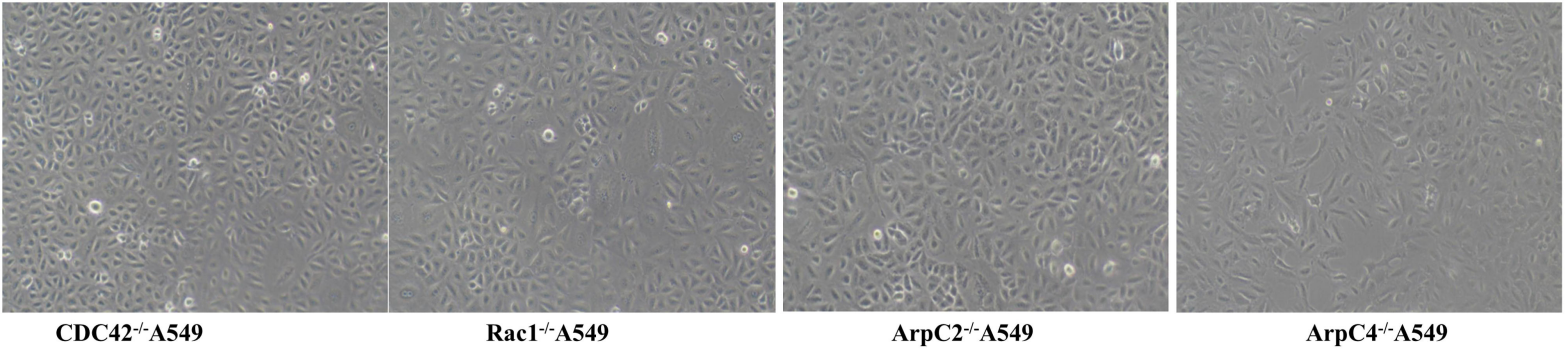
CDC42^-/-^ A549, Rac1^-/-^ A549, ArpC2^-/-^ A549 and ArpC4^-/-^ A549 cell lines.

The human alveolar type II epithelial cell line A549 was purchased from the Cell Resource Center, Institute of Basic Medical Sciences, Chinese Academy of Medical Sciences (1101HUM-PUMC000002). CRISPR-Cas9 knockout of CDC42^-/-^ A549 cells, Rac1^-/-^ A549 cells, ArpC2^-/-^ A549 cells and ArpC4^-/-^ A549 cells were achieved via electroporation using dual-sgRNA pairs designed to excise critical genomic regions, ensuring frameshifts and complete loss-of-function. For all resulting monoclonal lines (CDC42^-^/^-^, Rac1^-^/^-^, ArpC2^-^/^-^, ArpC4^-^/^-^), successful knockout was rigorously confirmed by Sanger sequencing, which unambiguously identified the intended large-fragment deletions or indels. This direct genomic validation circumvents potential ambiguities of protein-based methods, such as persistent protein detection due to long half-life or antibody specificity issues in Western blotting. The morphology of the cells after recovery revealed no discernible impact of genetic knockout on cellular architecture. Detailed results are presented in **Figure 1**.

All five cell lines were cultured in F-12K medium supplemented with 10% fetal bovine serum (FBS), 100 μg/mL penicillin and streptomycin, and incubated at 37 °C in a humidified 5% CO_2_ incubator. Culture medium was replaced every 1–2 days. Upon reaching 95%–100% confluence, cells were detached using 0.25% trypsin-EDTA, and experiments were conducted with cells at passages 3–5.

### Invasion test

Wild-type A549 cells, CDC42^-/-^ A549 cells, Rac1^-/-^ A549 cells, ArpC2^-/-^ A549 cells and ArpC4^-/-^A549 cells were inoculated in 6-well culture plates respectively, with a density of 2.5×10^5^ cells/well. Cells were infected with *M. catarrhalis* at an appropriate multiplicity of infection (MOI) of 10:1 (2.5×10^6^ bacteria/well) for 4 hours. Unattached bacteria were removed by washing with phosphate-buffered saline (PBS; pH 7.2–7.4). To eliminate extracellular bacteria, cells were treated with 300 μg/mL gentamicin for 1 hour, followed by PBS washes to remove residual antibiotic. Cells were detached with 800 μL 0.25% trypsin (Sigma) for 10 minutes, and then lysed with 1200 μL 0.1% saponin (Sigma) for 10 minutes. The lysates were transferred to 1.5 mL Eppendorf tubes and homogenized by repeated inversion. Serial 10-fold dilutions of lysates were prepared. Both undiluted and diluted lysates (100 μL) were plated onto 5% sheep blood agar plates for the number of internalized bacteria. Each dilution was plated in quadruplicate technical replicates, and experiments were repeated in three independent biological replicates.

### Transmission electron microscopy

Wild-type A549 cells, CDC42^-/-^ A549 cells, Rac1^-/-^ A549 cells, ArpC2^-/-^ A549 cells and ArpC4^-/-^A549 cells were seeded into 6-well cell culture plates at a density of 2.5×10^5^ cells/well. The plates were incubated at 37 °C in a humidified 5% CO_2_ incubator for 24 hours. Each cell line was assigned to three experimental groups: blank control, 73-OR strain infection, and ATCC-25238 strain infection. *M. catarrhalis* suspension (2.5×10^6^ CFU/well, MOI 10:1) was added to the designated wells. After 4 hours of co-cultivation, cells were washed twice with phosphate-buffered saline (PBS) and detached using 0.25% trypsin at 37 °C until mild detachment occurred. The enzymatic reaction was terminated by adding complete culture medium. The detached cells were transferred to 1.5 mL Eppendorf tubes and centrifuged. After discarding the supernatant, the cells were fixed with 2.5% glutaraldehyde for 48 hours. Fixed samples were processed for TEM. The intracellular and extracellular distribution of *M. catarrhalis* in A549 cells was analyzed. Additionally, the macropinocytosis-mediated engulfment of bacteria and the volume of resultant macropinosomes were compared across different experimental groups.

### Immunofluorescence and imaging

Wild-type A549 cells, CDC42^-/-^ A549 cells, Rac1^-/-^ A549 cells, ArpC2^-/-^ A549 cells and ArpC4^-/-^A549 cells were respectively seeded into 96-well plates at a density of 2×10^4^ cells/well. Subsequently, *M. catarrhalis* suspension was added to infect the cells at a multiplicity of infection (MOI) of 10:1 for 4 hours. After infection, cells were fixed with 4% paraformaldehyde in phosphate-buffered saline (PBS) at room temperature for 20 minutes. The solution was aspirated, and cells were washed 2–3 times with PBS. Permeabilization was performed using 0.1% Triton X-100 in PBS under dark conditions for 30 minutes, followed by three PBS washes. Then, cells were incubated with 50 μL/well of staining solution containing phalloidin (for F-actin labeling) and Hoechst 33342 (for nuclear staining) in PBS at room temperature for 1 hour in the dark. After removing the staining solution, cells were washed twice with PBS, and 200 μL of PBS was added to each well for imaging.

Fluorescence imaging was performed using a Perkin Elmer high-content imaging system (Operetta CLS; PerkinElmer, Hamburg, Germany). The DAPI channel (excitation/emission: 360/460 nm) was utilized to detect Hoechst 33342-labeled nuclei, while the FITC channel (excitation/emission: 488/520 nm) captured phalloidin-stained F-actin. Quantitative analysis of nuclear morphology (count, area) and F-actin content (fluorescence intensity, area) was conducted using dedicated analysis software (Harmony 4.9; PerkinElmer, Hamburg, Germany).

### F-Actin/G-Actin *In vitro* analysis

The F-Actin/G-Actin *In Vivo* Assay Kit (Catalog #BK037; Cytoskeleton, Inc., Denver, CO, USA) was employed to quantitatively assess the ratio of globular actin (G-actin) to filamentous actin (F-actin). Cultured cells were divided into two groups: (1) control group treated with culture medium, and (2) experimental group infected with *M. catarrhalis* strains 73-OR and ATCC-25238 at MOI of 10:1. Cells were lysed using a F-actin-stabilizing lysis buffer to preserve the structural integrity of F-actin while solubilizing G-actin. The lysate was subjected to sequential centrifugation. First centrifugation at 1,000×*g* for 5 minutes at 25 °C to remove cellular debris; Second centrifugation for the supernatant was further centrifuged at 100,000×*g* to separate insoluble F-actin (pellet) from soluble G-actin (supernatant).

Proteins were resolved via 10% sodium dodecyl sulfate-polyacrylamide gel electrophoresis (SDS-PAGE) and transferred onto polyvinylidene difluoride (PVDF) membranes for Western blotting. Anti-actin monoclonal antibody (Clone 7A8.2.1, Catalog #AAN02; Cytoskeleton, Inc.) was used for immunodetection, ensuring specificity for both G-actin and F-actin isoforms. Actin bands were quantified using densitometric scanning. The F-actin/G-actin ratio was calculated based on the integrated optical density (IOD) values of the corresponding bands. Intensities of protein bands in Western blots were determined with Image J (National Institutes of Health) (17).

### Statistical analysis

Statistical analysis was conducted using GraphPad Prism 5 software (GraphPad Software, San Diego, CA, USA). Statistical comparisons between two groups were performed using the unpaired Student’s t-test for normally distributed data or the Mann-Whitney U test for non-normally distributed data. Western blot results were analyzed using ImageJ 1.53i (National Institutes of Health, Bethesda, MD, USA). A statistically significant diffe*rence was defined as P<0.05.*

### Ethics statement

No human subjects were involved in this study. The study was approved by the Human Research Ethics Committee of the Peking Union Medical College (No. I-23PJ2153).

## Results

### Rho GTPases pathway involved in the polymerization/dissociation of actin in A549 cells

**Figure 2:**
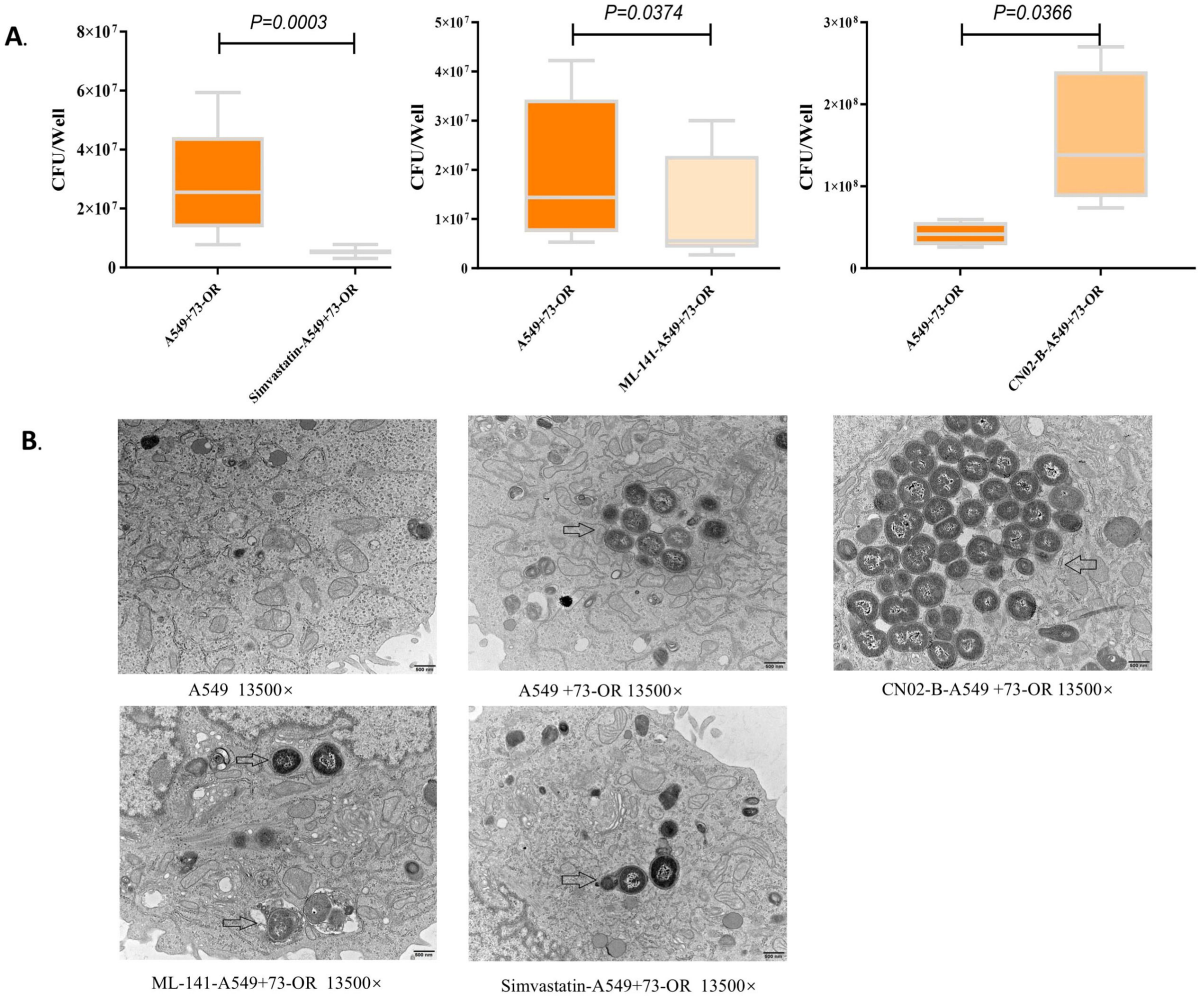
Effect of different inhibitors and activators on the invasion process of *M. catarrhalis* into A549. **(A)** Effects of different inhibitors and activators on the number of *M. catarrhalis* invading A549 cells; **(B)** Effects of different inhibitors and activators on the number of *M. catarrhalis* invading A549 cells under electron microscopy Black arrows indicate invading 73-OR (highly invasive *M. catarrhalis*).

This study investigated the involvement of key factors in the Rho GTPase signaling pathway regulating actin polymerization/depolymerization in A549 cells. Among them, A549 cells were pretreated with Arp2/3 complex inhibitor CK-636, CDC42 GTPase inhibitor ML 141, actin polymerization inhibitor Latrunculin A, Rho GTPase inhibitor Simvastatin, and Rac1 and CDC42 activator CN02-B. The untreated A549 cells served as the control group. Then, all groups were infected with the hypervirulent *M. catarrhalis* strain 73-OR. By invasion assay, the Rho GTPase pathway was confirmed to mediate *M. catarrhalis* internalization into A549 cells (**Figure 2A**). TEM revealed that CN02-B induced larger endosomal compartments after *M.catarrhalis* invaded A549 cells, suggesting enhanced actin-driven membrane remodeling. Conversely, the endosome volumes in the cells pretreated with Simvastatin and ML 141 were smaller. Simvastatin and ML 141 resulted in smaller endosomes, indicative of suppressed actin polymerization and impaired vesicle expansion (**Figure 2B**).

### Critical role of CDC42 in the Rho GTPase signaling pathway modulates the invasion of *M. catarrhalis* by regulating actin polymerization/depolymerization

**Figure 3:**
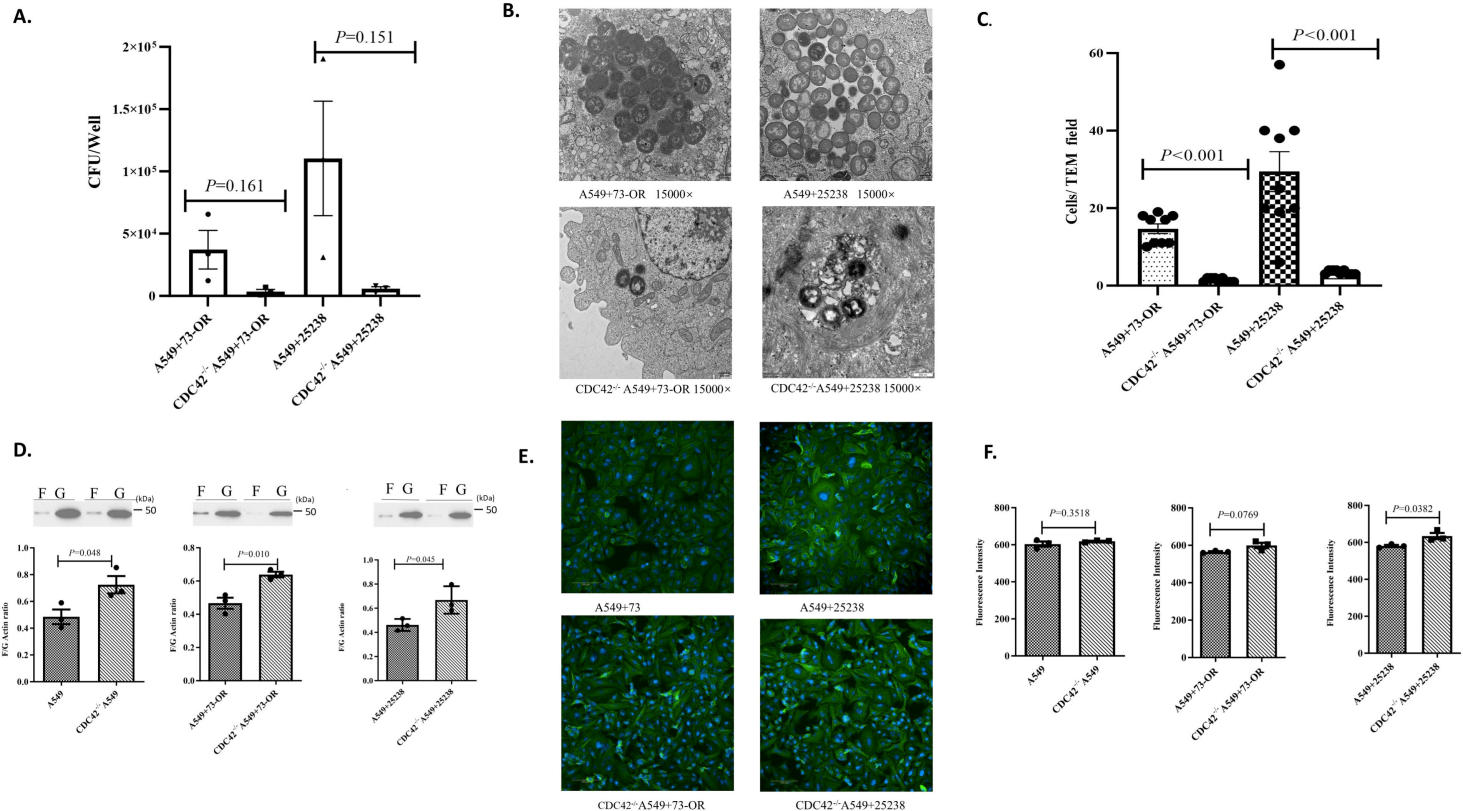
Critical role of CDC42 in the Rho GTPase signaling pathway during *M. catarrhalis* invasion into A549 cells. **(A)** Comparison of invasion counts of *M. catarrhalis* strains 73-OR and ATCC 25238 into WT A549 cells vs. CDC42^-/-^ A549 cells. Data are presented as mean ± standard error (SE) from three independent biological replicates (n=3). Statistical significance was determined using an unpaired Student’s t-test. To accurately assess the magnitude of the impact given the limited sample size, effect sizes (Cohen’s d) and mean differences are reported alongside P-values. **(B)** Transmission electron micrographs (TEM) of 73-OR and ATCC 25238 invading WT A549 cells and CDC42^-/-^ A549 cells. **(C)** TEM quantification of *M. catarrhalis* invasion into wild-type and CDC42^-/-^A549 cells. Invasion counts for *M. catarrhalis* strains 73-OR and ATCC 25238 were quantified by counting the number of bacteria internalized within wild-type (WT) and CDC42^-/-^ A549 cells via Transmission Electron Microscopy (TEM). Data represent the mean ± standard error(SE) from three independent biological replicates (n =3). Within each biological replicate, three random microscopic fields at the same magnification were selected and counted. Statistical significance between groups was determined using the non-parametric Mann-Whitney U test. Both 73-OR and ATCC 25238 strains showed a significant reduction in invasion into CDC42^-/-^ A549 cells compared to WT cells. **(D)** Quantitative comparison of F-actin/G-actin ratios in WT A549 cells and CDC42^-/-^ A549 cells post-infection with *M. catarrhalis* strains 73-OR and ATCC 25238. Data are presented as the mean ± standard error(SE) from three independent biological replicates (n=3). Statistical comparison between groups was performed using an unpaired Student’s t-test. **(E)** Immunofluorescence analysis of F-actin polymerization in A549 cells post-infection. Wild-type (WT) and CDC42^-/-^A549 cells were challenged with *M. catarrhalis* strains 73-OR and ATCC 25238, fixed, and stained. Blue: nuclei; Green: F-actin. **(F)** Analysis of microfilament expression levels in WT A549 cells vs. CDC42^-/-^ A549 cells following infection with 73-OR and ATCC 25238. Data are presented as the mean ± standard error(SE) from three independent biological replicates (n=3). Statistical comparison between groups was performed using an unpaired Student’s t-test.

In this study, we investigated whether CDC42, a key factor in the Rho GTPase signaling pathway, regulated actin polymerization/depolymerization dynamics and thereby modulated invasive efficiency of *M. catarrhalis*. The invasion assay showed the bacterial counts of 73-OR and ATCC 25238 strains invading CDC42^-/-^ A549 cells were significantly reduced compared to wild-type A549 cells (**Figure 3A**). For the 73-OR strain, invasion counts were dramatically reduced in CDC42^-/-^ A549 cells (Mean:3,510 ± 1,790 CFU/well) compared to wild-type A549 cells (Mean: 37,145 ± 15,501 CFU/well). This represents a mean difference of 33,635 ± 15,604 CFU/well. While the P-value was 0.161, the analysis yielded a very large effect size (Cohen’s d = 1.760), strongly indicating a highly potent biological dependence of 73-OR invasion on CDC42. Similarly, for the ATCC 25238 strain, the invasion reduction was even more profound (Mean Difference: 104,712 ± 46,045 CFU/well), with counts dropping from 110,433 ± 46,016 CFU/well in wild-type A549 cells to only 5,722 ± 1,639 CFU/well in CDC42^-/-^ A549 cells. Although the calculated P-value was 0.151, the effect size analysis again demonstrated a very large magnitude (Cohen’s d = 1.857), confirming that the invasive efficiency of both *M. catarrhalis* strains is highly dependent on CDC42 activity. Furthermore, TEM results directly show that CDC42^-/-^ A549 cells exhibited fewer internalized bacteria and smaller macropinosomes compared to wild-type A549 cells post-infection with both *M. catarrhalis* strains (**Figures 3B, C**). In addition, that CDC42^-/-^ A549 cells showed a significantly increased F-actin/G-actin ratio following bacterial infection compared to wild-type A549, indicating impaired actin depolymerization (**Figure 3D**). At the same time, by analyzing the fluorescence expression levels of microfilaments after infection of A549 cells by *M. catarrhalis*, the results also proved that compared with wild-type A549 cells infected by *Moraxella catarrhalis*, the expression level of microfilaments in CDC42^-/-^ A549 cells increased (**Figures 3E, F**). Thereby, CDC 42 modulates *M. catarrhalis* invasion by regulating actin cytoskeletal remodeling, as evidenced by reduced bacterial uptake, smaller macropinosomes, and elevated microfilaments in CDC42-deficient cells.

### Critical role of Rac1 in the Rho GTPase signaling pathway modulates the invasion of *M. catarrhalis* by regulating actin polymerization/depolymerization

**Figure 4:**
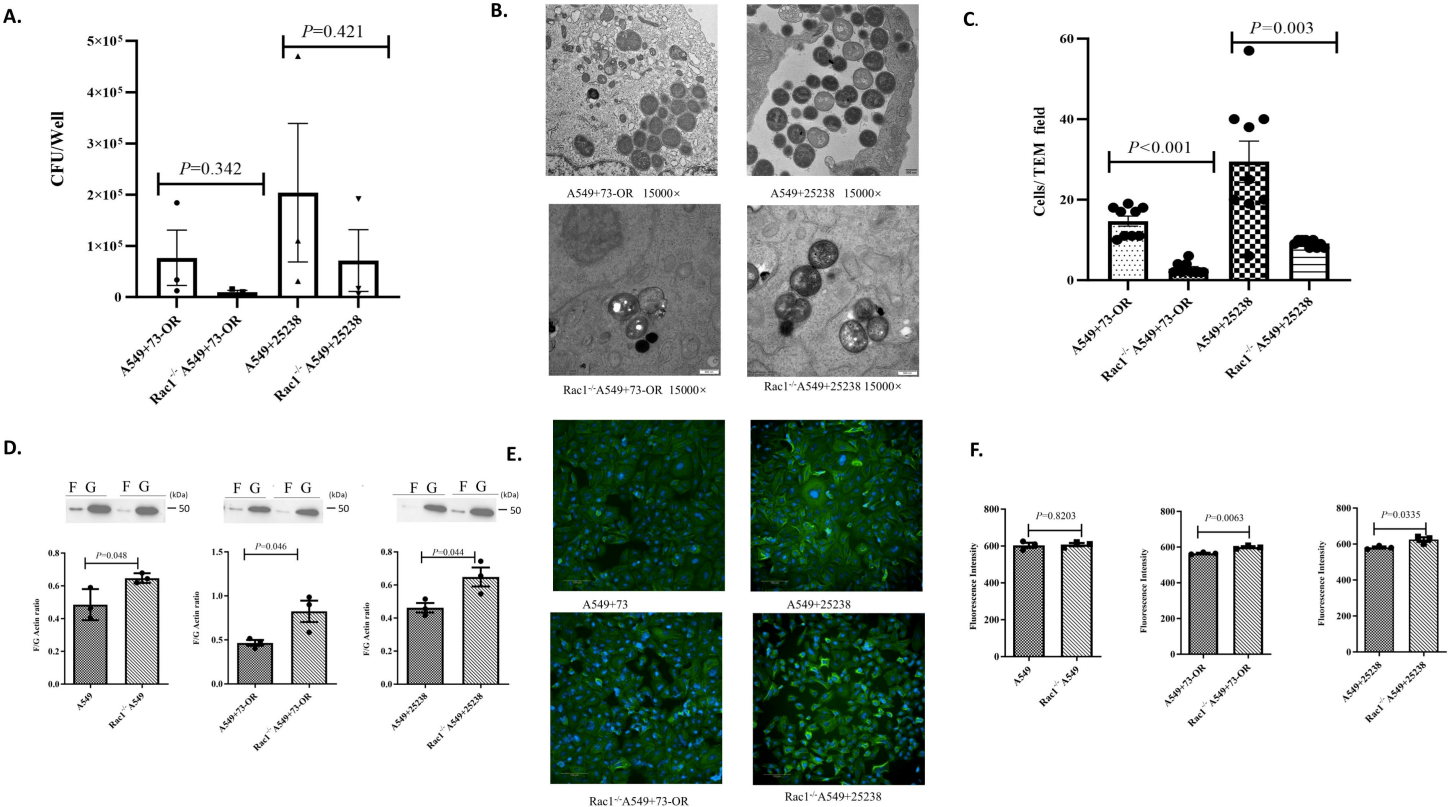
Critical role of Rac1 in the Rho GTPase signaling pathway during *M. catarrhalis* invasion into A549 cells. **(A)** Comparison of invasion counts of *M. catarrhalis* strains 73-OR and ATCC 25238 into WT A549 cells vs. Rac1^-/-^ A549 cells. Data are presented as mean ± standard error (SE)from three independent biological replicates (n=3). Statistical significance was determined using an unpaired Student’s t-test. To accurately assess the magnitude of the impact given the limited sample size, effect sizes (Cohen’s d) and mean differences are reported alongside P-values. **(B)** Transmission electron micrographs (TEM) of 73-OR and ATCC 25238 invading WT A549 cells and Rac1^-/-^ A549 cells. **(C)** TEM quantification of *M. catarrhalis* invasion into wild-type and Rac1^-/-^ A549 cells. Invasion counts for *M. catarrhalis* strains 73-OR and ATCC 25238 were quantified by counting the number of bacteria internalized within wild-type (WT) and Rac1^-/-^ A549 cells via Transmission Electron Microscopy (TEM). Data represent the mean ± standard error(SE) from three independent biological replicates (n=3). Within each biological replicate, three random microscopic fields at the same magnification were selected and counted. Statistical significance between groups was determined using the non-parametric Mann-Whitney U test. Both 73-OR and ATCC 25238 strains showed a significant reduction in invasion into Rac1^-/-^ A549 compared to WT cells. **(D)** Quantitative comparison of F-actin/G-actin ratios in WT A549 cells and Rac1^-/-^ A549 cells post-infection with *M. catarrhalis* strains 73-OR and ATCC 25238. Data are presented as the mean± standard error(SE) from three independent biological replicates (n=3). Statistical comparison between groups was performed using an unpaired Student’s t-test. **(E)** Immunofluorescence analysis of F-actin polymerization in A549 cells post-infection.Wild-type (WT) and Rac1^-/-^ A549 cells were challenged with *M. catarrhalis* strains 73-OR and ATCC 25238, fixed, and stained. Blue: nuclei; Green: F-actin. **(F)** Analysis of microfilament expression levels in WT A549 cells vs. Rac1^-/-^ A549 cells following infection with 73-OR and ATCC 25238. Data are presented as the mean ± standard error(SE) from three independent biological replicates (n=3). Statistical comparison between groups was performed using an unpaired Student’s t-test.

In this research section, we investigated whether Rac1, a pivotal component of the Rho GTPase signaling pathway, regulates actin polymerization/depolymerization dynamics and thereby modulates invasive efficiency of *M. catarrhalis*. Among them, the invasion assay results showed that compared with wild-type A549 cells, the number of bacteria invading Rac1^-/-^ A549 cells by 73-OR and ATCC 25238 decreased significantly (**Figure 4A**); For the 73-OR strain, invasion counts were substantially lower in Rac1^-/-^ A549 cells (Mean:9,833 ± 3,436 CFU/well) compared to WT A549 cells (Mean: 76, 562 ± 53, 978 CFU/well). While the P-value was 0.342, failing to reach conventional statistical significance, the analysis of the mean difference (66,728 ± 54, 087 CFU/well) revealed a large effect size (Cohen’s d=1.007), suggesting a high biological requirement for Rac1 in 73-OR internalization. Similarly, for the ATCC 25238 strain, invasion counts dropped from 203,750 ± 135,232 CFU/well in WT A549 cells to 71,173 ± 60,217 CFU/well in Rac1^-/-^ A549 cells (Mean Difference:132,577 ± 148,033 CFU/well). The difference was not statistically significant (P = 0.421),but the associated effect size (Cohen’s d = 0.731) indicates a moderate to large biological effect of Rac1 depletion on the invasive process. Also, the transmission electron microscopy results could directly show that the number of bacteria invading Rac1^-/-^ A549 cells by 73-OR and ATCC 25238 was significantly reduced, and the volume of macropinosomes formed was smaller (**Figures 4B, C**). Moreover, the results of detecting the expression levels of F-actin/G-actin indicated that compared with wild-type A549 cells infected by *M. catarrhalis*, the expression ratio of F-actin/G-actin in Rac1^-/-^ A549 cells was increased (**Figure 4D**). Meanwhile, by analyzing the fluorescence expression levels of microfilaments after infection of A549 cells by *M. catarrhalis*, the results also proved that compared with wild-type A549 cells infected by *M. catarrhalis*, the expression level of microfilaments in Rac1^-/-^ A549 cells was increased (**Figures 4E, F**). Therefore, Rac1 modulates *M. catarrhalis* invasion by governing actin cytoskeletal dynamics, as evidenced by reduced bacterial uptake, diminished macropinosome formation, and increased microfilaments in Rac1^-/-^ A549 cells.

### The Rho GTPase signaling factor ArpC2 don’t regulate actin polymerization/depolymerization dynamics and modulate *M. catarrhalis* invasion

**Figure 5:**
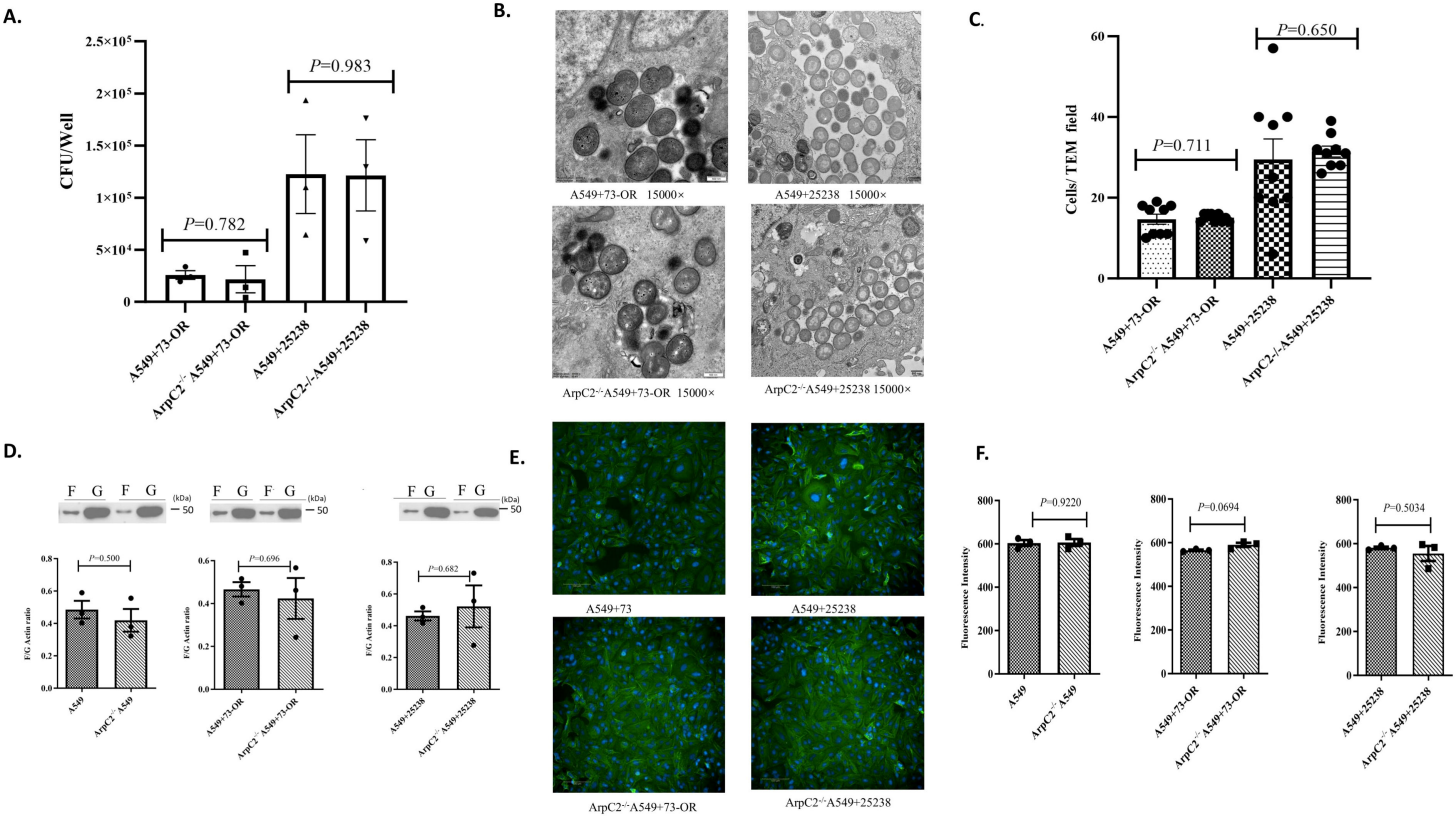
Critical role of ArpC2 in the Rho GTPase signaling pathway during *M. catarrhalis* invasion into A549 cells. **(A)** Comparison of invasion counts of *M. catarrhalis* strains 73-OR and ATCC 25238 into WT A549 cells vs. ArpC2^-/-^ A549 cells. Data are presented as mean ± standard error (SE)from three independent biological replicates (n=3). Statistical significance was determined using an unpaired Student’s t-test. To accurately assess the magnitude of the impact given the limited sample size, effect sizes (Cohen’s d) and mean differences are reported alongside P-values. **(B)** Transmission electron micrographs (TEM) of 73-OR and ATCC 25238 invading WT A549 cells and ArpC2^-/-^ A549 cells. **(C)** TEM quantification of *M. catarrhalis* invasion into wild-type and ArpC2^-/-^ A549 cells. Invasion counts for *M. catarrhalis* strains 73-OR and ATCC 25238 were quantified by counting the number of bacteria internalized within wild-type (WT) and ArpC2^-/-^ A549 cells via Transmission Electron Microscopy (TEM). Data represent the mean ± standard error(SE) from three independent biological replicates (n =3). Within each biological replicate, three random microscopic fields at the same magnification were selected and counted. Statistical significance between groups was determined using the non-parametric Mann-Whitney U test. **(D)** Quantitative comparison of F-actin/G-actin ratios in WT A549 cells and ArpC2^-/-^ A549 cells post-infection with *M. catarrhalis* strains 73-OR and ATCC 25238. Data are presented as the mean ± standard error(SE) from three independent biological replicates (n=3). Statistical comparison between groups was performed using an unpaired Student’s t-test. **(E)** Immunofluorescence analysis of F-actin polymerization in A549 cells post-infection. Wild-type (WT) and ArpC2^-/-^A549 cells were challenged with M. catarrhalis strains 73-OR and ATCC 25238, fixed, and stained. Blue: nuclei; Green: F-actin. **(F)** Analysis of microfilament expression levels in WT A549 cells vs ArpC2^-/-^ A549 cells following infection with 73-OR and ATCC 25238. Data are presented as the mean ± standard error(SE) from three independent biological replicates (n=3). Statistical comparison between groups was performed using an unpaired Student’s t-test.

Through this study, we investigated whether ArpC2, a critical component of the Rho GTPase signaling pathway, modulates invasive efficiency of *M. catarrhalis* by regulating actin polymerization/depolymerization dynamics. The invasion assay suggested bacterial counts of *M. catarrhalis* strains 73-OR and ATCC 25238 invading ArpC2^-/-^ A549 cells showed no significant difference compared to wild-type A549 cells (**Figure 5A**). The invasion assay suggested that bacterial counts for both *M. catarrhalis* strains invading ArpC2^-/-^A549 cells showed no significant difference compared to wild-type A549 cells. For the 73-OR strain, invasion counts were 25,700 ± 4,197 CFU/well in the WT group and 21,653 ± 13,043 CFU/well in the knockout group (Mean Difference: 4,047 ± 13,701 CFU/well). This minor reduction was highly non-significant (P = 0.782), with a negligible effect size (Cohen’s d = 0.241), suggesting ArpC2 is not a primary factor regulating this process. Similarly, for the ATCC 25238 strain, the results were even more pronounced, with invasion remaining almost identical between the WT group (122,533 ± 37,828 CFU/well) and the ArpC2^-/-^ group (121,350 ± 34,178 CFU/well). The difference was statistically negligible (P = 0.983), corresponding to a trivial effect size (Cohen’s d = 0.019). TEM results could directly show that the number of bacteria invading ArpC2^-/-^ A549 cells by 73-OR and ATCC 25238 was not reduced, and the volume of the macropinosomes formed was larger (**Figures 5B, C**). Additionally, F-Actin/G-Actin *in vivo* analysis indicated no significant alteration in the F-actin/G-actin ratio was detected between infected wild-type A549 and ArpC2^-/-^ A549 cells (**Figure 5D**). Consistent with the above results, F-actin expression levels in ArpC2^-/-^ cells remained significantly unchanged compared to wild-type A549 controls following bacterial infection (**Figures 5E, F**). Thus, ArpC2 did not participate in regulating the polymerization/dissociation process of actin and subsequently modulating the number of *M. catarrhalis* invading A549 cells.

### The key Rho GTPase signaling factor ArpC4 regulates actin polymerization/depolymerization dynamics but don’t modulate *M. catarrhalis* invasion

**Figure 6:**
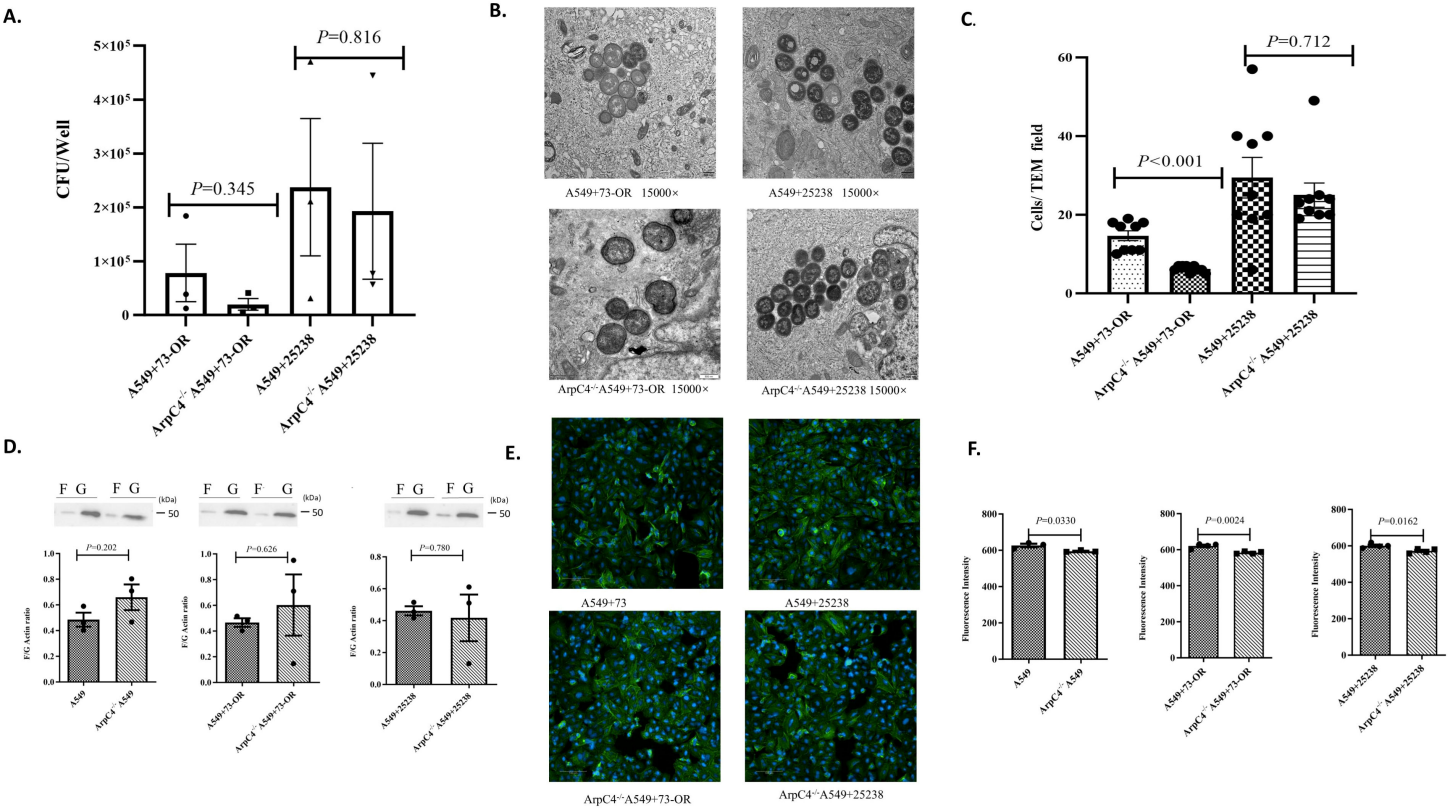
Critical role of ArpC4 in the Rho GTPase signaling pathway during *M. catarrhalis* invasion into A549 cells. **(A)** Comparison of invasion counts of *M. catarrhalis* strains 73-OR and ATCC 25238 into WT A549 cells vs. ArpC4^-/-^ A549 cells. Data are presented as mean ± standard error (SE)from three independent biological replicates (n=3). Statistical significance was determined using an unpaired Student’s t-test. To accurately assess the magnitude of the impact given the limited sample size, effect sizes (Cohen’s d) and mean differences are reported alongside P-values. **(B)** Transmission electron micrographs (TEM) of 73-OR and ATCC 25238 invading WT A549 cells and ArpC4^-/-^ A549 cells. **(C)** TEM quantification of *M. catarrhalis* invasion into wild-type and ArpC4^-/-^ A549 cells. Invasion counts for *M.catarrhalis* strains 73-OR and ATCC 25238 were quantified by counting the number of bacteria internalized within wild-type (WT) and ArpC4^-/-^ A549 cells via Transmission Electron Microscopy (TEM). Data represent the mean ± standard error(SE) from three independent biological replicates (n=3). Within each biological replicate, three random microscopic fields at the same magnification were selected and counted. Statistical significance between groups was determined using the non-parametric Mann-Whitney U test. **(D)** Quantitative comparison of F-actin/G-actin ratios in WT A549 cells and ArpC4^-/-^ A549 cells post-infection with *M. catarrhalis* strains 73-OR and ATCC 25238. Data are presented as the mean ± standard error(SE) from three independent biological replicates (n=3). Statistical comparison between groups was performed using an unpaired Student’s t-test. **(E)** Immunofluorescence analysis of F-actin polymerization in A549 cells post-infection. Wild-type (WT) and ArpC4^-/-^A549 cells were challenged with *M. catarrhalis* strains 73-OR and ATCC 25238, fixed, and stained. Blue: nuclei; Green: F-actin. **(F)** Analysis of microfilament expression levels in WT A549 cells vs ArpC4^-/-^ A549 cells following infection with 73-OR and ATCC 25238. Data are presented as the mean ± standard error(SE) from three to four independent biological replicates (n=3-4). Statistical comparison between groups was performed using an unpaired Student’s t-test.

We investigated whether ArpC4, an essential factor of the Rho GTPase signaling pathway regulates actin polymerization/depolymerization dynamics and thereby modulates invasive efficiency of *M. catarrhalis*. Both *M. catarrhalis* strains 73-OR and ATCC 25238 invading ArpC4^-/-^ A549 cells demonstrated no significant difference compared to wild-type A549 cells. The invasion assay suggested that bacterial counts for both *M. catarrhalis* strains invading ArpC4^-/-^A549 cells demonstrated no significant difference compared to wild-type A549cells. However, detailed analysis showed distinct trends. For the 73-OR strain, invasion counts were notably reduced in ArpC4^-/-^ A549 cells (Mean: 19,990 ± 10,832 CFU/well) compared to WT cells (Mean: 78,212 ± 53,344 CFU/well). While this difference did not reach statistical significance(P = 0.345), the analysis revealed a large effect size (Cohen’s d = 0.873) suggesting a strong biological tendency for ArpC4 to regulate 73-OR internalization. In contrast, for the ATCC 25238 strain, the invasion counts remained nearly identical between WT cells (237,300 ± 127,533 CFU/well) and ArpC4^-/-^cells (192,750 ± 126,182 CFU/well). The difference was statistically negligible (P = 0.816), corresponding to a trivial effect size (Cohen’s d=0.203). (**Figure 6A**). Electron microscopy showed that the number of 73-OR and ATCC 25238 invading ArpC4^-/-^ A549 cells was reduced, and the size of the macropinosomes formed was large (**Figures 6B, C**). While ArpC4^-/-^ A549 cells showed altered F-actin/G-actin ratios under basal conditions, no infection-dependent modulation was detected (**Figure 6D**). The analysis of the fluorescence expression of microfilaments in A549 cells infected with *M. catarrhalis* also proved that ArpC4 may affect the expression of microfilaments, but it is not related to *M. catarrhalis* infection (**Figures 6E, F**). The above results indicate that ArpC4 participates in the regulation of actin cytoskeletal dynamics but does not contribute to *M. catarrhalis* invasion into A549 cells, as evidenced by unaltered bacterial uptake and infection-independent actin stabilization.

## Discussions

During the pathogenic process, *M. catarrhalis* interacts with host respiratory epithelial cells during infection, triggering immune responses via extracellular and intracellular receptors (18). A critical invasion mechanism involves macropinocytosis, a non-clathrin, non-caveolin-dependent endocytic process driven by actin polymerization under specific stimuli. Macropinocytosis mediates the uptake of macromolecules, viruses, and bacteria into macropinosomes. This process relies on actin-rich membrane ruffling to form protrusions (e.g., pseudopodia, circular ruffles, or blebs), which encapsulate extracellular fluid and pathogens before detaching from the plasma membrane (19). Our previous studies demonstrated that *Moraxella catarrhalis* invasion into A549 cells leads to reduced F-actin expression compared to wild-type A549 cells. However, among different bacterial strains, higher F-actin levels correlate with increased invasion efficiency (12). Previous studies primarily focused on pathogen variability to explain differences in invasion. In contrast, this study aims to elucidate the underlying regulatory mechanisms by maintaining a fixed bacterial strain and instead modifying host cellular pathways, specifically targeting the Rho GTPases signaling pathway. Experimental evidence confirms that, under constant bacterial conditions, host cells autonomously regulate actin polymerization/depolymerization to modulate *M. catarrhalis* internalization. The Rho GTPases pathway and its associated factors bidirectionally regulate actin dynamics, thereby indirectly influencing bacterial endocytosis. Our findings confirm the central role of actin dynamics during *Moraxella catarrhalis* invasion. However, the novelty of this study is primarily demonstrated in the following two aspects: First, we not only confirmed the overall significance of Rho GTPase signaling but also precisely identified and distinguished specific regulators (e.g., CDC42 and Rac1) as critical factors modulating *M. catarrhalis* invasion of host respiratory epithelial cells, and determined whether additional components (ArpC2 and ArpC4) directly participate in actin polymerization. Furthermore, our data elucidate a more specific mechanism by providing novel insights into how this pathogen modulates the host cytoskeleton through specific key factors. Thus, our discoveries represent a critical complement and refinement to the understanding of Rho GTPase signaling pathways.

Rho GTPases, small GTP-binding proteins in eukaryotes, involved in the transmission of various important cellular regulatory signals. They are classified into 8 subfamilies: classical (Rho, Rac, CDC42, RhoD/F) and non-classical (Rnd, RhoBTB, RhoH, RhoU/V). These proteins share high sequence homology and can bind to G protein-coupled receptors, tyrosine kinase receptors, cytokine receptors or adhesion molecule receptors to activate downstream signaling pathways (20). Members of the Rho GTPases family are closely related to various biological behaviors such as cell morphology, polarity, cell adhesion and migration, and play a crucial role in various cellular life activities by regulating processes such as actin polymerization/depolymerization, membrane receptor binding and membrane fold formation (20, 21). The regulation of actin polymerization by Rho GTPases is a very complex process that requires the involvement of multiple factors. Among them, Rac1-Pak1/Pak2 and Rac1-IRSp53-Wave2 activate Arp2/3 to generate branched actin networks and lamellipodia (22, 23). CDC42-Rac1-MRCK phosphorylates myosin phosphatase targeting subunit (MYPT), enabling ​WASP-mediated Arp2/3 activation and membrane protrusion (13, 15). Arp 2/3 can bind to actin monomers and play a template role, causing intracellular actin monomers to assemble into microfilaments. As new formed actin branches grow, the plasma membrane is squeezed out, causing the membrane to extend into pseudopods (15). In addition to forming cell structures, it also involved in the formation of cell-cell junction assembly, the movement of pathogens and vesicle trafficking, etc. (24). In our study, we knocked out CDC42 and Rac1 in A549 cells respectively to reduce the endogenous Rho GTPases level in A549 cells, and then observed the differences in the number of bacteria invading wild-type A549 cells and knockout cells, as well as the volume of macropinocytosis and the expression level of microfilaments during the invasion process. The invasion of host cells by bacteria is mediated through the Rho GTPases signaling pathway, which regulates actin polymerization and depolymerization. During bacterial invasion, the host initiates a defensive state to resist further pathogen entry, characterized by decreased F-actin expression and reduced polymerization (12). In Rho GTPases knockout cells, bacteria lose the ability to manipulate actin dynamics via this pathway, resulting in attenuated invasion efficiency and the loss of F-actin suppression. Notably, within this signaling pathway, both CDC42 and Rac1 are involved in dual regulatory roles, coordinating bacterial internalization as well as actin polymerization/depolymerization.

Arp2/3 is a critical regulator of actin polymerization. It binds to actin monomers to generate branched actin networks, which play essential roles in vesicle trafficking, endocytosis/exocytosis, and cell motility (25). Arp2/3-dependent actin polymerization/depolymerization also drives invasive movements of various pathogens (26). This complex comprises seven evolutionarily conserved subunits: two actin-related protein subunits (Arp2 and Arp3) and five auxiliary subunits (ArpC1, ArpC2, ArpC3, ArpC4, and ArpC5). Arp2 and Arp3 form the core of the complex, adopting a C-shaped structure, while the other subunits stabilize the complex through peripheral interactions (27). The subunits ArpC2 and ArpC4 share similar spatial architectures, characterized by an α/β fold composed of alpha-helices and beta-sheets. Both subunits bind laterally to the mother actin filament, facilitating branch formation through nucleation of a new daughter filament (28). During bacterial invasion of host cells, actin polymerization is either directly induced by the pathogens or mediated via cytoskeletal state-associated signaling molecules, leading to cytoskeletal rearrangement that facilitates bacterial entry and intracellular motility. In infections caused by *Clostridium* species, enteropathogenic *Escherichia coli* (EPEC), and Gram-positive bacilli, the Arp2/3 complex subunits ArpC2 and ArpC4 are implicated in bacterial adhesion, host cell invasion, and the initiation of inflammatory responses (29–31). In this study, we analyzed the bacterial quantity and microfilament expression during the invasion of *M. catarrhalis* into wild-type A549 cells and ArpC2^-/-^ A549 cells, and found that ArpC2 did not participate in the actin polymerization/depolymerization process of *M. catarrhalis* invading A549 cells and did not affect the quantity of *M. catarrhalis* invading cells. However, the study of *M. catarrhalis* invading ArpC4^-/-^ A549 cells revealed that ArpC4 might affect microfilament expression, but did not change the quantity of *M. catarrhalis* invading A549 cells or the volume of phagolysosomes. This result confirmed that ArpC4 participates in regulating the actin polymerization/depolymerization process, but does not regulate the invasion of *M. catarrhalis*. The conformational changes and functional roles of each subunit of Arp2/3 in the process of actin polymerization and microfilament formation are complex (32). Meanwhile, there are few studies on the specific mechanism of how Arp2/3 members regulate actin polymerization/depolymerization during pathogen invasion of host cells. Therefore, further research is needed to help us understand the effects and mechanisms of each subunit of Arp2/3 on the functions of pathogen infection-related host cells.

This study has some limitations. The sample size used in this study was limited. The *M. catarrhalis*. quality control strain ATCC-25238 and the representative strain with strong invasion ability 73-OR should be included in future studies to confirm the universality of these findings. Secondly, this study only confirmed the regulatory role of the Rho GTPase signaling pathway in *M. catarrhalis* invading A549 cells *in vitro*. Future studies should include more isolates and even animal experiments. In addition, most proteins of Rho GTPases convert between the inactive GDP-bound form and the active GTP-bound form. Activated GTPases interact with downstream effector proteins to stimulate diverse cellular signaling pathways. In this study, the signaling molecules involved in the activation of Rho GTPases during *M. catarrhalis* invading host cells were not explained. This will also become the focus of our subsequent research.

In conclusion, the study clarified that key factors Rac1 and CDC42 of the Rho GTPase signaling pathway modulate the invasion of *M. catarrhalis* into host cells by regulating actin polymerization/depolymerization. ArpC4 participates in regulating the actin polymerization/depolymerization process but does not regulate the invasion of *M. catarrhalis*; however, ArpC2 does not participate in regulating the actin polymerization/depolymerization process or the invasion of *M. catarrhalis*. This research provides important theoretical basis for the prevention or treatment of AECOPD caused by *M. catarrhalis* in the future through innate immunity and other means.

## Data Availability

The original contributions presented in the study are included in the article/supplementary material. Further inquiries can be directed to the corresponding author/s.
